# Comparing Canada’s 2018 proposed and 2022 final front-of-pack labelling regulations using generic food composition data and a nationally representative dietary intake survey

**DOI:** 10.1017/S1368980024001496

**Published:** 2024-10-29

**Authors:** Jennifer J Lee, Christine Mulligan, Mavra Ahmed, Mary R L’Abbé

**Affiliations:** 1 Department of Nutritional Sciences, Temerty Faculty of Medicine, University of Toronto, Toronto, ON, M5S 1A8, Canada; 2 Joannah & Brian Lawson Centre for Child Nutrition, University of Toronto, Toronto, ON, M5S 1A8, Canada

**Keywords:** Food labelling, Nutrition labelling, Food policy, Front-of-pack labelling, Nutrient intakes

## Abstract

**Objective:**

The objective of the study was to compare the potential dietary impact of proposed and final front-of-pack labelling (FOPL) regulations (published in *Canada Gazette I (CG1)* and *Canada Gazette II (CG2)*, respectively) by examining the difference in the prevalence of foods that would require a ‘High in’ front-of-pack nutrition symbol and nutrient intakes from those foods consumed by Canadian adults.

**Design:**

Foods in a generic food composition database (*n* 3676) were categorised according to the details of FOPL regulations in *CGI* and *CGII*, and the differences in the proportion of foods were compared. Using nationally representative dietary survey data, potential intakes of nutrients from foods that would display a ‘High in’ nutrition symbol according to *CGI* and *CGII* were compared.

**Setting:**

Canada

**Participants:**

Canadian adults (≥ 19 years; *n* 13 495)

**Results:**

Compared with *CGI*, less foods would display a ‘High in’ nutrition symbol (Δ = –6 %) according to *CGII* (saturated fat = –4 %, sugars = –1 %, sodium = –3 %). Similarly, potential intakes of nutrients-of-concern from foods that would display a ‘High in’ nutrition symbol were reduced according to *CGII* compared with *CGI* (saturated fat = –21 %, sugars = –2 %, sodium = –6 %). Potential intakes from foods that would display a ‘High in’ nutrition symbol were also reduced for energy and nutrients-to-encourage, including protein, fibre, calcium and vitamin D.

**Conclusions:**

Changes to FOPL regulations may have blunted their potential to limit intakes of nutrients-of-concern; however, they likely averted potential unintended consequences on intakes of nutrients-to-encourage for Canadians (e.g. calcium and vitamin D). To ensure policy objectives are met, FOPL regulations must be monitored regularly and evaluated over time.

Non-communicable diseases are one of the major causes of disability and pre-mature mortality globally and in Canada,^(1)^ and unhealthy diet is one of the major preventable risk factors^(2)^. Front-of-pack labelling (FOPL), which uses a simple and easy-to-understand symbol displayed on the front of food packages to communicate the healthfulness of the food^(3)^, has been shown to be an effective public health strategy to improve dietary intakes of a population^(4)^. FOPL has been shown to promote manufacturer-driven product reformulation^(5)^ and influence individual dietary behaviours at the point-of-purchase^(3,6)^. Following the implementation of the Chilean Food Labelling and Advertising Law (which included mandatory FOPL for foods ‘high in’ energy, sugars, saturated fat or sodium), the proportion of products displaying a ‘high in’ front-of-pack label decreased from 51 % to 44 %^(5)^. Similarly, household purchases of calories and nutrients-of-concern were lower in the post-policy period compared with the pre-policy purchasing trends^(7)^. In addition to the results from these naturalistic observational studies in Chile, experimental studies from multiple countries have also shown that front-of-pack labels or symbols, particularly those that highlight ‘high’ levels of nutrients-of-concern, decreased purchasing intentions of foods with those nutrients^(8,9)^.

In 2016, Canada introduced the *Healthy Eating Strategy* to improve the dietary patterns and the food environment for Canadians by ‘*making the healthier choice, the easier choice*’^(10)^. The *Healthy Eating Strategy* consisted of a suite of voluntary initiatives and mandatory regulations, including updating Voluntary Sodium Reduction Targets, amendments to food labelling regulations and introducing FOPL regulations^(10)^. Canadian FOPL regulations were promulgated in 2022, requiring pre-packaged food and beverage products meeting and/or exceeding thresholds for nutrients-of-concern to display a ‘High in’ nutrition symbol by January 2026^(11)^. FOPL regulations target three nutrients-of-concern (i.e. saturated fat, sugars and sodium) based on the excessive intakes of these nutrients among Canadians^(12–14)^ associated with adverse health outcomes.

Six years were taken to finalise FOPL regulations from the initial conception of the policy idea (i.e. the introduction in the *Healthy Eating Strategy*) to the publication of the final FOPL regulations in 2022. The draft FOPL regulations were first published in *Canada Gazette I* (*CGI*) in 2018^(15)^ following initial stakeholder consultations. After 4 years of additional research and public consultations on *CGI*, final FOPL regulations were published in July 2022 in *Canada Gazette II* (*CGII*)^(11)^. The target nutrients-of-concern remained the same between FOPL regulations in *CGI* (i.e. draft) and *CGII* (i.e. final); however, several aspects of the FOPL regulations in *CGII* differed from those published in *CGI*, including changing thresholds for foods with small reference amounts and broadening of the exemption criteria. Previous studies examining policy development activities in Canada^(16)^ and globally^(17,18)^ have reported a pattern of industry-affiliated lobbyists attempting to hinder or weaken FOPL regulations. However, limited studies have examined the potential dietary impact resulting from regulatory changes in food and nutrition policies, which can provide insights into how comments received and lobbying efforts during the regulatory development process can influence policy outcomes. Therefore, the objective of the study was to compare Canadian FOPL regulations according to *CGI* and *CGII* by examining the difference in the proportion of foods that would need to display a ‘High in’ nutrition symbol and the potential nutrient intakes from those foods by Canadian adults.

## Methods

### Canadian Front-of-Pack Labelling regulations

Table 1 shows a summary of the main components of Canadian FOPL regulations published in *CGI* and *CGII*. Online supplementary material, Supplemental Table 1 shows nutrient thresholds of FOPL regulations published in *CGI* and *CGII*.

**Table 1:** Summary of the proposed (*Canada gazette I*) and final front-of-pack labelling regulations (*Canada gazette II*) in Canada

	Proposed FOPL Regulations (as published in *Canada Gazette I,* 2018)	Final FOPL Regulations (as published *in Canada Gazette II*, 2022)
Nutrients-of-concern	• Saturated fat • Sugars (total) • Sodium	• Saturated fat • Sugars (total) • Sodium
Exemption criteria	1) Foods that have shown health benefits • Examples: unflavoured milk, small serving size foods with high unsaturated fat content 2) Foods exempted from displaying NFt • Examples: fresh fruits and vegetables, single ingredient meats, foods sold in very small packages 3) Foods that are well-known sources of nutrients-of-concern • Examples: honey, salt, syrup	1) Health-related exemptions: • Foods that have shown health protective benefits • *Full exemptions* for all 3 nutrients-of-concern (i.e. never display a ‘High in’ nutrition symbol); examples: all unflavoured milk from any animals, eggs, fruits and vegetables, oils, nuts, fatty fish • *Conditional exemptions* for some nutrients-of-concern (i.e. may display a ‘High in’ nutrition symbol for non-exempted nutrient(s)); examples: exemptions for saturated fat and sodium for foods that are important sources of ‘shortfall nutrients,’ such as cheese and yogurt products ‘high in’ calcium^‡^ • *Conditional exemptions* if sole sources(s) of nutrients-of-concern are one of the exempted foods listed above; example: exemptions for saturated fat for tuna in olive oil 2) Technical-related exemptions: • Foods exempted from displaying NFt • Examples: fresh fruits and vegetables, single-ingredient meats, foods sold in very small packages • Includes single-ingredient ground meats, as nutrition composition is similar to freshly cut meats exempted from displaying NFt 3) Practical-related exemptions: • Well-known sources of nutrients-of-concern • Examples: honey, salt, butter
Threshold levels, overview	• Foods with a reference amount < 50 g or ml containing ≥ 5 %DV of nutrient per reference amount: ≥ 15 %DV per 50 g or ml of the food • Foods with a reference amount ≥ 50 g or ml: ≥ 15 % DV per reference amount • Meals and main dishes with a reference amount ≥ 200 g: ≥ 30 % DV per reference amount	• Foods with a reference amount ≤ 30 g or ml: ≥ 10 % DV per reference amount • Foods with a reference amount > 30 g or ml: ≥ 15 % DV per reference amount • Main dishes with a reference amount ≥ 200 g^†^: ≥ 30 % DV per reference amount
Reference amount	• 2016 Table of Reference Amounts for Food • 24 major and 173 minor categories	• 2022 Table of Reference Amounts for Food • 24 major, 185 minor and 3 sub-categories
Daily values	• Published in 2016 • *Saturated fat:* 10 g (1–< 4-year-olds) and 20 g for all others • *Sugars (total):* 50 g (1–< 4-year-olds) and 100 g for all others • Sodium*:* 1500 mg (1–< 4-year-olds) and 2300 mg for all others	• Published in 2022 (for implementation by January 1, 2026) • *Saturated fat:* 10 g (1–< 4-year-olds) and 20 g for all others • *Sugars (total):* 50 g (1–< 4-year-olds) and 100 g for all others • Sodium*:* 1200 mg (1–< 4-year-olds) and 2300 mg for all others
‘High in’ nutrition symbol(s)	*Four symbols were proposed:* 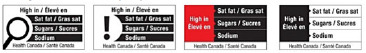	Final symbol was selected: 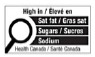

Details of the proposed and final FOPL regulations can be found in *Canada Gazette I* ^(15)^ and *Canada Gazette II* ^(11)^, respectively. *Specific threshold levels are shown in online supplementary material, Supplemental Table 1. ^†^For main dishes for children 1 to < 4 years of age, reference amount of 170 g is used instead of 200 g. ^‡^‘High-in’ calcium is defined as ≥ 10 %DV per reference amount for foods with a reference amount ≤ 30 g or ml; and ≥ 15 %DV per reference amount for foods with a reference amount > 30 g or ml. Abbreviations: %DV, Percent Daily Value; FOPL, Front-of-pack Labelling; NFt, Nutrition Facts table.

Briefly, draft FOPL regulations in *CGI* would have mandated pre-packaged foods to display a ‘High in’ front-of-pack nutrition symbol for meeting and/or exceeding threshold levels for nutrients-of-concern (saturated fat, total sugars and sodium)^(15)^. Threshold levels would have been set using percent daily value (%DV)^(19)^, updated in 2016, per reference amount for each nutrient-of-concern based on the food type (i.e. foods and meals/main dishes) and age groups (i.e. children ≥ 4 years of age and adults and 1 to < 4-year-old children). For foods with small reference amounts (i.e. < 50 g or ml) that contain ≥ 5 %DV of nutrients-of-concern per reference amount (according to Health Canada’s 2016 Table of Reference Amounts for Food (TRA)^(20)^ or serving size reported on Nutrition Facts table (NFt), whichever is greater), nutrient levels of the food would have been assessed using the reference amount of 50 g (or mL) of the food to identify concentrated sources of saturated fat, sugars and/or sodium. The thresholds would have been set as 15 %DV for all foods and 30 %DV for all meals and main dishes (defined as foods with reference amounts ≥ 200 g). Foods meeting any of the three exemption criteria would not have displayed a ‘High in’ nutrition symbol regardless of their levels of nutrients-of-concern^(15)^. The three exemption criteria included foods that have shown health benefits (e.g. unflavoured milk, eggs, fruits and vegetables), foods that are exempted from displaying an NFt (e.g. fresh fruits and vegetables, single ingredient meat, foods with small package sizes) and foods that are known sources of the target nutrients-of-concern (e.g. honey, syrup, salt).

Final FOPL regulations in *CGII* mandate pre-packaged foods to display a ‘High-in’ front-of-pack nutrition symbol for meeting and/or exceeding threshold levels for target nutrients-of-concern (saturated fat, total sugars and sodium)^(11)^. The threshold levels using %DV^(21)^, updated in 2022, are set based on the age group (i.e. 1 to < 4-years-old and all ages ≥ 4-years-old) and the reference amount of food for each nutrient. The reference amount is determined by whichever is greater between the revised 2022 TRA^(22)^ or the serving size presented on the NFt. The thresholds are 10 %DV for foods with small reference amounts (i.e. ≤ 30 g or ml), 15 %DV for all other foods (i.e. reference amounts > 30 g or ml) and 30 %DV for main dishes (defined as combination dishes with reference amounts ≥ 200 g or ≥ 170 g, if designed for 1 to < 4-year-old children)^(11)^. There are three exemption criteria for foods that would not display a ‘High in’ nutrition symbol regardless of the levels of nutrients-of-concern: health-, technical-, and practical-related exemptions^(11)^. Health-related exemption criteria apply to foods that have a recognised health protection benefit, including all unflavoured milk, eggs, fruits, vegetables, oils high in unsaturated fats, cheese and yogurt products ‘high in’ calcium, and any multi-ingredient foods with sole sources of nutrients-of-concern contributing to their levels (e.g. diced fruits packed in water would be exempted from displaying a ‘High in’ nutrition symbol for sugars, canned tuna in olive oil would be exempted from displaying a ‘High in’ nutrition symbol for saturated fat). Technical-related exemptions apply to foods that are exempted from displaying an NFt, including fresh fruits and vegetables, single-ingredient meats, foods sold in very small packages and foods sold at farmer’s market. Practical-related exemptions apply to foods that are known sources of the target nutrients, including honey, syrup, salt and butter.

In addition, the table of DV used in nutrition labelling was updated in October 2022 for implementation by January 1, 2026^(21)^, with lower daily values for sodium for foods intended for children 1 to < 4 years of age (i.e. 1500 mg *v*. 1200 mg). The updated daily values were used for assessment only against *CGII* thresholds, as these values will be applied when FOPL regulations are implemented in 2026. TRA published in 2016^(20)^ was revised in November 2022^(22)^ with updates to reference amounts and descriptions of some categories and the addition of new categories. 2016 TRA category consisted of 24 major and 173 minor categories, while 2022 TRA category consisted of 24 major, 185 minor and 3 sub-categories. 2016 TRA was used to categorise foods according to *CGI,* and revised TRA 2022 was used to categorise foods according to *CGII* to apply the most relevant TRA categories at the time of the publication of each version of the FOPL regulations.

### Food composition database

The Canadian Nutrient File (CNF) 2015, a generic food composition database of 6904 commonly consumed foods by Canadians, was used in this analysis. The CNF contains information on over 150 nutrients, derived from the United States Department of Agriculture National Nutrient Database for Standard Reference, adapted to reflect Canadian fortification levels, regulatory standards and specific Canadian food items^(23)^. Free sugar levels, not readily available in the CNF, were estimated by Wang et al.^(24)^. Food missing levels of saturated fat, sugars and/or sodium were considered to have negligible amounts of these nutrients (*n* 357). All foods in the CNF were classified by 2016 TRA^(20)^ and 2022 TRA^(22)^ categories.

### Food level analysis

Among foods in the CNF, meals created using common preparation methods reported by Canadians (*n* 3169) were excluded as individual ingredients were included in the analysis; and foods that are not subject to FOPL regulations (e.g. alcoholic beverages, nutritional supplements; *n* 59) were excluded from the analysis. The final analytic sample was 3676. Foods were categorised based on the total number of nutrients-of-concern a food meets and/or exceeds thresholds (i.e. exempted from regulations, no ‘High in’ nutrition symbol, and display a ‘High in’ nutrition symbol for 1–3 nutrients) and the type of nutrients-of-concern it exceeds (i.e. saturated fat, sugars, sodium) according to *CGI* and *CGII*; the proportion of foods categorised according to *CGI* and *CGII* was compared based on the FOPL categories, as previously reported^(25)^.

### Dietary data

Data from nationally representative, cross-sectional dietary survey (Canadian Community Health Survey (CCHS)–Nutrition 2015) were used in this study, described in detail elsewhere^(26)^. Briefly, the CCHS-Nutrition 2015 collected 24-hour dietary recall and a general health questionnaire survey data from 20 487 Canadians, selected using a clustered sampling method based on Canadian census data to ensure a sample representative of the Canadian population in terms of age, sex, geography and socio-economic status^(26)^. A subsample was invited and completed a second 24-hour dietary recall (*n* 7623). The CCHS-Nutrition 2015 included data from individuals > 1 year living in private dwellings in the 10 Canadian provinces and excluded data from full-time members of the Canadian Forces or those who live in the Territories, on reserves and other Indigenous settlements, in some remote areas or institutions^(26)^.

Similar to a previous study assessing the potential intakes of nutrients-of-concern according to *CGII* ^(27)^, only the first 24-hour dietary recall data from adults were used in this analysis (≥ 19 years). Data were excluded from respondents < 19 years of age (*n* 6568), underweight (BMI < 18·5 kg/m^2^; *n* 230), lactating (*n* 183) or from individuals reporting no food consumption (i.e. only non-caloric foods or special dietary foods excluded from FOPL regulations; *n* 11), resulting in a final sample size of 13 495.

Misreporters of energy intake (EI) were identified using the ratio of reported EI to estimate total energy expenditure (TEE)^(28)^. TEE was calculated based on age, sex, measured BMI (or corrected self-reported BMI using the Statistics Canada’s correction factor^(29)^) and physical activity levels (i.e. sedentary, low active, moderately active and highly active) using the Institute of Medicine equations^(30)^. In the absence of anthropometric information, TEE was estimated using age, sex and physical activity levels as reported in the Dietary Guidelines for Americans 2020–2025^(31)^. Misreporters were defined as those with EI:TEE ratio < 0·7 (i.e. under-reporters) or > 1·42 (i.e. over-reporters); all other respondents were defined as plausible reporters (i.e. EI:TEE ratio 0·7-1·42)^(28)^.

### Dietary level analysis

Foods reported in the CCHS-Nutrition 2015 were matched to foods in the CNF database by Statistics Canada^(26)^. The first-day 24-hour recall data from this CNF-matched CCHS-Nutrition 2015 were linked to the research team’s CNF database with foods classified according to *CGI* and *CGII* to estimate the intakes of nutrients and energy according to the FOPL regulation categories. Potential intakes of nutrients-of-concern targeted by FOPL regulations (i.e. saturated fat, total sugars and sodium), energy and other nutrients-of-public health interest, not subject to FOPL regulations (i.e. protein, free sugars, fibre, calcium and vitamin D), from foods categorised by *CGI* and *CGII* were examined. As FOPL regulations do not apply to foods sold in restaurants, intakes of nutrients and energy from ‘foods away from home,’ which are defined in CCHS-Nutrition 2015 as foods consumed outside of ‘home’ in a limited-service or full-service restaurant^(32)^, were categorised separately.

### Statistical analysis

All statistical analyses were performed using SAS version 9.4, (SAS Institute Inc., Cary, NC, USA). For the food level assessment, the number and proportion of foods in the CNF categorised according to *CGII* were calculated and compared with values categorised according to *CGI*, obtained from Mulligan et al.^(25)^, overall and by 2022 TRA major food categories. For the dietary level assessment, potential intakes of nutrients-of-concern (i.e. saturated fat, total sugars and sodium), energy and other nutrients-of-public health interest, not subject to FOPL regulations (i.e. nutrients-of-concern (free sugars) and nutrients-to-encourage (protein, fibre, calcium and vitamin D)), from foods categorised according to *CGI* and *CGII*, and as foods away from home were calculated as a proportion to total intakes of each nutrient and energy. The balanced repeated replication technique with 500 replicates using bootstrap weights and sample survey weights provided by Statistics Canada was applied to obtain representative population-level estimates appropriate for the CCHS-Nutrition 2015 survey design. Potential intakes of nutrients and energy were adjusted for potential confounders (age, sex, EI (except for saturated fat and sugars, as intakes were expressed as a proportion to total EI) and misreporting status (i.e. under-, plausible- and over-reporters)). Potential intakes of nutrients-of-concern, energy and free sugars from foods categorised according to *CGII* were obtained from Lee et al.^(27)^. One-way repeated measures ANOVA was used to evaluate the statistical difference between the potential intakes of nutrients-of-concern, energy and nutrients-of-public health interest from foods categorised according to *CGI* and *CGII.* To account for the large sample size falsely detecting statistical significance of small differences in the proportion of potential intakes, statistical significance was set at *P* < 0·001.

## Results

### Food-level analysis

Table 2 shows the difference in the number and proportion of foods that would display a ‘High in’ nutrition symbol according to *CGI* and *CGII* (i.e. proposed and final, respectively) overall and by TRA major category; and online supplementary material, Supplemental Table 2 shows the number and proportion of foods that would display a ‘High in’ nutrition symbol according to *CGII* overall and by TRA major category. According to *CGII*, 30 % of foods would display a ‘High in’ nutrition symbol (*n* 1105/3676), which is 6 % less (Δ = –211) than foods that would have needed to display a ‘High in’ nutrition symbol according to *CGI*. The difference is due to an increased number of foods that would meet the exemption criteria according to *CGII* (*n* 341, 9·3 % overall). Among TRA categories, the biggest change was seen in the *Dairy Products and Substitutes* food category, where 33 % of the foods in this category (Δ = –69) that needed to display a ‘High in’ nutrition symbol according to *CGI* would not need to display a ‘High in’ nutrition symbol according to *CGII*. Overall, the proportion of foods that would display a ‘High in’ nutrition symbol for exceeding threshold levels decreased by 4 % for saturated fat (Δ = –133), by 1 % for total sugars (Δ = –40) and by 3 % for sodium (Δ = –95).

**Table 2:** Difference in the number and proportion of food and beverage products that would display a ‘High in’ nutrition symbol according to the proposed (*Canada gazette I*) and final (*Canada gazette II*) front-of-pack labelling regulations

TRA Category	‘High in’ nutrition symbol	%	Exempted	%	No ‘High in’ nutrition symbol (< thresholds)	%	Nutrient-of-concern type on a ‘High in’ nutrition symbol					
							Saturated fat	%	Sugars (total)	%	Sodium	%
A. Bakery products	−19	–7·5 %	0		+19	7·5 %	0		−11	–4·3 %	−11	–4·3 %
B. Beverages	0		0		0		0		0		0	
C. Cereals and other grains	+6	3·2 %	0		−6	–3·2 %	+3	1·6 %	+2	1·1 %	+5	2·7 %
D. Dairy products and substitutes	−69	–33·2 %	20	9·6 %	+49	23·6 %	−58	–27·9 %	−7	–3·4 %	−43	–20·7 %
E. Desserts	0		0		0		0		0		0	
F. Dessert Toppings and fillings	0		0		0		0		0		0	
G. Eggs and substitutes	−2	–9·5 %	+2	+9·5 %	0		−2	–9·5 %	0		−1	–4·8 %
H. Fats and oils	−31	–21·5 %	+95	+66·0 %	−64	–44·4 %	−21	–14·6 %	0		−8	–5·6 %
I. Seafood and substitutes	−27	–10·8 %	+108	+43·0 %	−81	–32·3 %	−9	–3·6 %	0		−19	–7·6 %
J. Fruits and fruit juices	−2	–0·6 %	0		2	0·6 %	0		−2	–0·6 %	0	
K. Legumes	0		0		0		0		0		0	
L. Meats and substitutes	−34	–3·9 %	+40	+4·6 %	−6	–0·7 %	−36	–4·2 %	0		−7	–0·8 %
M. Miscellaneous	−2	–2·5 %	0		2	2·5 %	0		−2	–2·5 %	−2	–2·5 %
N. Combination Dishes	0		0		0		+1	5·3 %	0		0	
O. Nuts and Seeds	−6	–6·3 %	+69	+72·6 %	−63	–66·3 %	−5	–5·3 %	−1	–1·1 %	0	
P. Potatoes	−2	–7·4 %	+4	+14·8 %	−2	–7·4 %	−1	–3·7 %	0		−2	–7·4 %
R. Sauces and Dips	−6	–8·5 %	0		6	8·5 %	0		−1	–1·4 %	−5	–7·0 %
S. Snacks	−1	–1·9 %	+2	+3·7 %	−1	–1·9 %	−1	–1·9 %	0		0	
T. Soups	0		0		0		0		0		0	
U. Sugars and Sweets	−11	–10·8 %	0		+11	10·8 %	−3	–2·9 %	−15	–14·7 %	0	
V. Vegetables	−4	–1·0 %	+1	+0·3 %	+3	0·8 %	0		−2	–0·5 %	−2	–0·5 %
W. Foods for < 4 years old	−1	–1·0 %	0		+1	1·0 %	−1	–1·0 %	−1	–1·0 %	0	
Overall total	−211	–5·7 %	+341	9·3 %	−130	–3·5 %	−133	–3·6 %	−40	–1·1 %	−95	–2·6 %

*n* 3676. Number and proportion of foods in the Canadian Nutrient File were categorised according to final front-of-pack labelling regulations overall and by 2022 Table of Reference Amounts for Food (TRA) major category^(22)^), and compared with the number and proportion according to the proposed regulations (as reported in Mulligan et al.^(25)^).

### Dietary level analysis

**Fig. 1:**
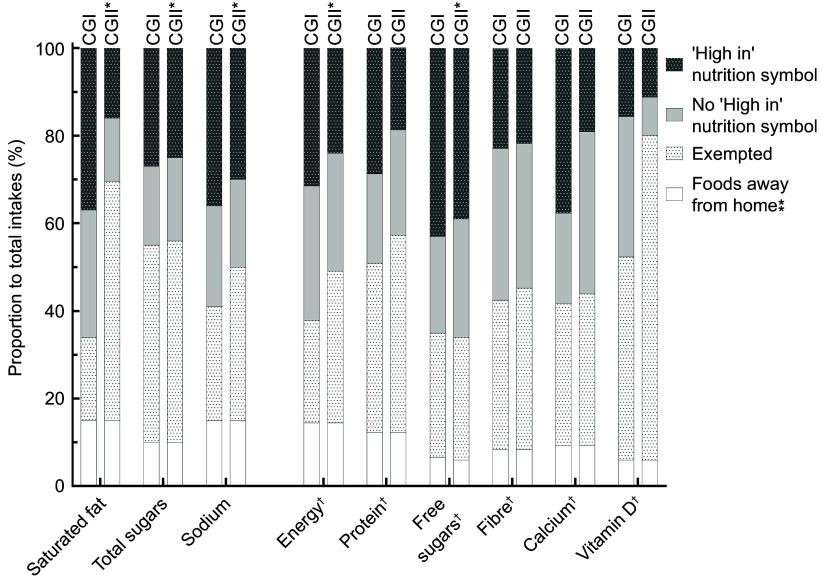
Proportion of potential intakes of nutrients and energy from foods categorised according to the proposed *(Canada Gazette I (CGI))* and final (*Canada Gazette II (CGI))* front-of-pack labelling (FOPL) regulations*. n* 13 495. Potential intakes from foods away from home, defined as foods consumed in a limited-service or full-service restaurant^(32)^, were categorised and analysed separately from other FOPL regulation categories. Intakes of nutrients and energy were estimated using the balanced repeated replication technique with 500 replicates to obtain representative population-level estimates and adjusted for potential confounders confounders (age, sex, energy intake (except for saturated fat and sugars, as intakes were expressed as a proportion to total energy intake), and misreporting status (i.e. under-, plausible- and over-reporters)). One-way repeated measures ANOVA was used to evaluate the difference between the potential intakes of nutrients and energy from foods categorised according to *CGI* and *CGII.* Statistical significance was set at *P* < 0·001. All potential intakes were significantly different (*P* < 0·001) except potential intakes of total and free sugars from that would be exempted from the regulations (*P* = 0·02 and *P* = 0·98, respectively). *Potential intakes from foods categorised according to *CGII* were obtained from Lee et al.^(27)^. ^†^As levels of energy and other nutrients-of-public health concern are not subject to FOPL regulations, intakes from exempted foods referred to foods meeting or exceeding the exemption criteria for all three nutrients-of-concern. Therefore, foods categorised under “No ‘High in’ nutrition symbol” include products that may have conditional exemptions for specific nutrient(s)-of-concern (e.g. dairy products exempted for saturated fat or sodium only). Abbreviations: CG, Canada Gazette; FOPL, front-of-pack labelling.

Figure 1 shows the summary of the differences in the proportion of potential intakes of energy and nutrients from foods categorised according to *CGI* and *CGII*. Compared with *CGI*, the proportion of potential intakes of nutrients-of-concern from foods that would display a ‘High in’ nutrition symbol according to *CGII* was reduced for all (saturated fat: 37 % *v*. 16 %; total sugars: 27 % *v*. 25 %; sodium: 36 % *v*. 30 %; *P* < 0·001 for all). Similarly, compared with *CGI*, potential intakes of energy and other nutrients-of-public health interest, not subject to FOPL regulations, from foods that would display a ‘High in’ nutrition symbol according to *CGII* was reduced (energy: 32 % *v*. 24 %; protein: 29 % *v*. 19 %; free sugars: 43 % *v*. 39 %; fibre: 23 % *v*. 22 %; calcium: 38 % *v*. 19 % and vitamin D: 16 % *v*. 11 %). The difference in the proportion of potential intakes of energy and nutrients from foods categorised according to *CGI* and *CGII* were all significant (*P* < 0·001), except for potential intakes of total sugars (45 % *v*. 46 %, *P* = 0·02) and free sugars (28 % *v*. 28 %, *P* = 0·98) from foods that would be exempted from FOPL regulations.

## Discussion

Using a generic food composition database and nationally representative dietary intake survey data, we examined the potential dietary implications of changes to Canadian FOPL regulations during the regulatory development process. The expansion of the exemption criteria and the changes to nutrient thresholds resulted in fewer foods being required to display a ‘High in’ nutrition symbol according to final FOPL regulations, particularly for saturated fat and sodium. Although the difference in the proportion was relatively small at the food level (6 %), this difference translated to a significant reduction in the potential intakes of nutrients-of-concern (2–21 %) at the dietary level. However, potential intakes of nutrients-to-encourage from products that would display a ‘High in’ nutrition symbol, particularly calcium and vitamin D, were also significantly reduced as a result of changes to FOPL regulations. Although the intakes of nutrients-of-concern were blunted due to the regulatory changes, they likely prevented a ‘High in’ nutrition symbol from inadvertently discouraging consumers from choosing products that may be significant contributors of nutrients-to-encourage.

One of the key differences in FOPL regulations was the broadening of the exemption criteria to include cheese, yogurt and other dairy-related products ‘high in’ calcium, which likely contributed to the blunted potential impact that FOPL regulations could have on reducing intakes of saturated fat and sodium. Our findings show that the *Dairy Products and Substitutes* food category (i.e. TRA Category D) had the greatest influence on the overall proportion of foods that would display a ‘High in’ nutrition symbol, demonstrating the impact that changes to FOPL regulations have on cheese and yogurt products ‘High in’ saturated fat and/or sodium and calcium. At the dietary level, these changes translated to lower intakes of calcium and vitamin D from foods that would display a ‘High in’ nutrition symbol according to *CGII* compared with *CGI.* This is likely since dairy products (particularly milk) are the top sources of calcium and vitamin D among Canadians^(33,34)^. Considering the high prevalence of calcium and vitamin D inadequacies among Canadians^(35)^, our findings support the expansion of the exemption criteria of ‘high’ calcium dairy products to protect the potential impact that FOPL may have on intakes of these nutrients-to-encourage. However, considering cheese products are also significant contributors of saturated fat and sodium among Canadians^(36,37)^ with ‘high’ levels of saturated fat and/or sodium (as shown in the present study), the impact of FOPL regulations on nutrient intakes and the food supply must be closely monitored.

Other regulatory changes to the exemption criteria included the exemption of additional sources of foods ‘high in’ saturated fat, contributing to the greatest difference in the potential intakes of saturated fat from foods that would display a ‘High in’ nutrition symbol. According to final FOPL regulations in *CGII*, foods naturally ‘high in’ saturated fat, including ground meats (as they are nutritionally similar to freshly cut meats that meet the technical exemption criteria) and butter (as per the practical exemption criteria) are now exempt^(11)^. These exemption criteria are not consistent with the dietary guidelines of Canada’s food guide^(38)^ and the WHO guidelines on saturated fats^(39)^, which recommend limiting saturated intakes to < 10 % total EI. Canadian FOPL regulations, in its binary format (i.e. display a ‘High in’ nutrition symbol or not), will help Canadians easily identify most foods that are ‘high in’ nutrients-of-concern; however, they may not necessarily help Canadians differentiate the healthfulness of these exempted foods that would not display a ‘High in’ nutrition symbol. Israel, for example, has adopted a dual FOPL system, where ‘high in’ symbols were mandated to highlight foods high in nutrients-of-concern and a voluntary ‘Mediterranean diet’ symbol was implemented to highlight foods that align with the Mediterranean diet^(40)^. The United States is currently developing a voluntary front-of-pack ‘healthy’ symbol for the recently updated ‘healthy’ claim criteria^(41)^ aligned with the Dietary Guidelines for Americans 2020–2025. Our findings suggest additional public health strategies, including those that leverage ‘healthy’ dietary patterns, may be helpful to complement binary FOPL regulations to promote healthy dietary behaviours, beyond reducing intakes of nutrients-of-concern.

Another difference in FOPL regulations was the change in thresholds for small serving-size products, which likely had an impact on the potential intakes of sodium and total sugars. Proposed FOPL regulations in *CGI* standardised reference amounts to 50 g or ml of the food with small reference amounts (i.e. < 50 g or ml) to apply the 15 %DV thresholds, same as other foods with larger reference amounts of ≥ 50 g or ml; however, this was changed in *CGII* with a more liberal threshold of 10 %DV per reference amount without the standardisation. For example, barbeque sauce, a small serving-sized product, with 30 g of total sugars and 700 mg of sodium per 100 ml, would have needed to display a ‘High in’ nutrition symbol for both sugars and sodium according to *CGI* (15 g of total sugars/50 ml meets 15 %DV, and ∼350 mg of sodium/50 ml exceeds 15 %DV), but not according to *CGII* (9 g of total sugars/30 ml is below 10 %DV, and ∼210 mg of sodium/30 ml is below 10 %DV). This is particularly concerning for certain food categories, such as sauces and condiments, that have historically been poor at reformulating products to reduce the levels of nutrients-of-concern^(42)^. Although the consumption of these foods may be typically small, it does not dismiss the fact that these foods are good sources of nutrients-of-concern. Further, the 10 %DV threshold for small serving-sized foods is not consistent with other Canadian food labelling regulations, including the interpretative footnote on NFt (‘5 % is a little and 15 % is a lot’^(43)^) and nutrient criteria for health claims, which typically calculate thresholds per standardised reference amount of 50 g or ml of food^(44)^. For consistent messaging and to increase the potential dietary impact of FOPL regulations, re-examination of the current thresholds is highly encouraged.

Our findings revealed that the changes in final FOPL regulations (i.e. *CGII*) from the proposed regulations (i.e. *CGI*) blunted the potential impact that the regulations could have on the intakes of nutrients-of-concern and the rationale for all the changes has been poorly communicated to the public. After the announcement of the *Healthy Eating Strategy*, Health Canada commissioned consumer research and launched a public consultation to explore different aspects of the regulations. Research studies examined the effect of the proposed ‘High in’ nutrition symbols on consumer understanding (i.e. identify foods ‘high in’ nutrients-of-concern) and behavioural intentions (i.e. food choice)^(45)^ and explored various aspects of the ‘High in’ nutrition symbol related to noticeability (e.g. design, location and proximity to other food information)^(46,47)^. However, there is limited evidence on the potential impact of the regulatory development process of the nutrient profiling model underpinning FOPL regulations (i.e. exemption criteria, thresholds for nutrients-of-concern). Recent studies examining the lobbying registrations for the *Healthy Eating Strategy* reported about 40 % of the industry-affiliated corporations registered to lobby on FOPL regulations^(48)^, demonstrating the extent that lobbyists have attempted to hinder or weaken FOPL regulations in Canada. Although the changes to FOPL regulations may not have been solely related to industry lobbying, timely evaluation of public health policies and the reporting of the regulatory development process must be conducted to ensure they are based on scientific evidence that will support policy objectives and prioritise the health of a population; and such findings should be shared with the public to improve openness and transparency in the regulatory process.

We examined the potential dietary implications of changes to a food policy using a national generic food composition database and nationally representative dietary intake survey data, providing a comprehensive overview of the direct and indirect dietary impacts of changes that occurred during the regulatory development process. However, there are a few limitations to note. First, we used the generic food composition database (i.e. CNF) to examine the changes to FOPL regulations; however, the findings may not adequately evaluate the effectiveness of final FOPL regulations. The generic food composition database aggregates the nutrient levels of similar foods found in the market^(26)^, while FOPL regulations will influence each individual food, particularly pre-packaged foods, differently^(25)^. Future studies evaluating the impact of FOPL regulations using a branded food composition database (e.g. Food Label Information and Price^(49)^) will be needed to monitor and evaluate the effectiveness of the regulations on the pre-packaged food supply. Second, we only examined the potential impact on Canadian adults, but not children and adolescents, who may have different dietary patterns than adults^(50)^; therefore, they may be influenced differently by FOPL regulations. With a lack of studies examining the potential impact of FOPL regulations on children and adolescents, future studies are warranted to evaluate how FOPL regulations could influence the intakes of different subpopulations.

In conclusion, changes in FOPL regulations that occurred during the consultation phase of the regulatory process resulted in the blunting of the potential dietary impact of the final regulations by targeting fewer potential intakes of nutrients-of-concern. However, these regulatory changes also resulted in preserving the potential intakes of nutrients-to-encourage (e.g. calcium and vitamin D). Our findings highlight the complexity of developing food and nutrition policies, underscoring the need for robust monitoring and evaluation of these policies to ensure policy objectives are met and to continue to improve the diets of the target population.

## Supporting information

Lee et al. supplementary material 1 Lee et al. supplementary material

Lee et al. supplementary material 2 Lee et al. supplementary material

## Data Availability

The data underlying the results presented in the study are available from Statistics Canada: https://www150.statcan.gc.ca/n1/en/catalogue/82M0024X2018001. Analytic code can be made available upon request to the corresponding author.

## Supplementary material

For supplementary material accompanying this paper visit https://doi.org/10.1017/S1368980024001496
