# Supplementary material for: Comparing Canada’s 2018 proposed and 2022 final front-of-pack labelling regulations using generic food composition data and a nationally representative dietary intake survey
Source: Public Health Nutr. 2024 Oct 29;27(1):e223. doi: 10.1017/S1368980024001496 (PMC11604314; doi:10.1017/S1368980024001496)
Supplement: Lee et al. supplementary material 1 — Lee et al. supplementary material [file S1368980024001496sup001.docx]

**Supplementary Table 1.** Nutrient thresholds which would determine the display of a ‘High in’ front-of-pack nutrition symbol according to the proposed (*Canada Gazette I*) and final (*Canada Gazette II*) front-of-pack labelling regulations.

|  | **Proposed FOPL Regulations  (as published in *Canada Gazette I,* 2018)** | | | | **Final FOPL Regulations  (as published *in Canada Gazette II*, 2022)** | | | | | |  |
| --- | --- | --- | --- | --- | --- | --- | --- | --- | --- | --- | --- |
| **Category** | Children  1- to <4-year-old | Pre-packaged foods | Pre-packaged meals and main dishes^*^ | Foods with RA ≤30 g or 30 mL for  1- to <4-year-old children | | Foods with RA >30 g or 30 mL for  1- to <4-year-old children | Main dishes with RA  ≥170 g for 1- to <4-year-old children | Foods with RA ≤30 g or 30 mL for  ≥4-year-olds | Foods with RA >30 g or 30 mL for  ≥4-year-olds | Main dishes with RA ≥200 g for  ≥4-year-olds |  |
| **Thresholds, %DV** | | | | | | | | | | | |
|  | 15% | 15% | 30% | 10% | | 15% | 30% | 10% | 15% | 30% |  |
| **Thresholds, absolute amount per nutrient** | | | | | | | | | | | |
| Saturated fat (g) | 1.5 | 3 | 6 | 1 | | 1.5 | 3 | 2 | 3 | 6 |  |
| Total sugars (g) | 8^†^ | 15 | 30 | 5 | | 8^†^ | 15 | 10 | 15 | 30 |  |
| Sodium (mg) | 225^⁑^ | 350^†^ | 690 | 120^⁑^ | | 180^⁑^ | 360^⁑^ | 230 | 350^†^ | 690 |  |

Details of the proposed front-of-pack labelling (FOPL) regulations can be found in *Canada Gazette I^(1)^.* and the final regulations in *Canada Gazette II*^(2)^. According to *Canada Gazette I*, the levels of nutrients-of-concern are assessed based on the reference amount (as per Health Canada’s most up-to-date Table of Reference Amounts for Foods (TRA)), serving size (as shown on the Nutrition Facts table), or 50 g or 50 mL of the food (if the reference amount and serving size are <50 g or 50 mL, and the %DV of the nutrient-of-concern per reference amount or serving size is ≥5%), whichever is the greatest^(1)^. According to *Canada Gazette II*, the levels of nutrients-of-concern per reference amount (as per TRA^(3)^) or serving size (as shown on the Nutrition Facts table), the greater amount between the two, is used^(2)^. ^*^Pre-packaged meals and main dishes were defined as products in the *Combination Dishes* categories (TRA minor categories N.1 and N.2), including chicken and rice casserole, chili, pizza, salad kit/bowl consumed as a meal^(4)^. ^†^The values are adjusted according to the rounding rules for nutrition labelling information as per *Food and Drug Regulations^(5)^*. ^⁑^Table of daily values used in nutrition labelling were updated in October 2022 for implementation by 2025 with lower daily values for sodium for foods intended for children 1 to <4 years of age (i.e., 1,500 mg vs. 1,200 mg). The updated daily values (i.e., 1,200 mg) were used for assessment against *CGII* thresholds as these values will be applied when FOPL regulations are implemented in 2026. Abbreviations: %DV, Percent Daily Value; FOPL, front-of-pack labelling; RA, Reference Amount; TRA, Table of Reference amounts for Foods.

# Supplementary Table 2. Summary of the total number and proportion of foods that would display a nutrition symbol based on front-of-pack labelling regulations (*Canada Gazette II)* overall and by Table of Reference Amounts for Foods major category.

|  |  | **‘High in’ nutrition symbol** | | | | **No ‘High in’ nutrition symbol** | | |
| --- | --- | --- | --- | --- | --- | --- | --- | --- |
| **TRA Category^*^** | **n** | **1 Nutrient** | **2 Nutrients** | **3 Nutrients** | **Total** | **Exempted** | **<Thresholds** | **Total** |
| A. Bakery Products | 254 | 103 (40.6%) | 26 (10.2%) | 0 | 129 (50.8%) | 0 | 125 (49.2%) | 125 (49.2%) |
| B. Beverages | 81 | 29 (35.8%) | 3 (3.7%) | 7 (8.6%) | 39 (48.1%) | 0 | 42 (51.9%) | 42 (51.9%) |
| C. Cereals & Other Grains | 188 | 18 (9.6%) | 4 (2.1%) | 0 | 22 (11.7%) | 0 | 166 (88.3%) | 166 (88.3%) |
| D. Dairy Products & Substitutes | 208 | 63 (30.3%) | 22 (10.6%) | 1 (0.5%) | 86 (41.3%) | 32 (15.4%) | 90 (43.3%) | 122 (58.7%) |
| E. Desserts | 70 | 39 (55.7%) | 20 (28.6%) | 0 | 59 (84.3%) | 0 | 11 (15.7%) | 11 (15.7%) |
| F. Dessert Toppings & Fillings | 8 | 6 (75.0%) | 1 (12.5%) | 0 | 7 (87.5%) | 0 | 1 (12.5%) | 1 (12.5%) |
| G. Eggs & Substitutes | 21 | 0 | 0 | 1 (4.8%) | 1 (4.8%) | 16 (76.2%) | 4 (19.0%) | 20 (95.2%) |
| H. Fats & Oils | 144 | 31 (21.5%) | 4 (2.8%) | 0 | 35 (24.3%) | 95 (66.0%) | 14 (9.7%) | 109 (75.7%) |
| I. Seafood & Substitutes | 251 | 20 (8.0%) | 2 (0.8%) | 0 | 22 (8.8%) | 204 (81.3%) | 25 (10.0%) | 229 (91.2%) |
| J. Fruits & Fruit Juices | 343 | 124 (36.2%) | 0 | 0 | 124 (36.2%) | 148 (43.1%) | 71 (20.7%) | 219 (63.8%) |
| K. Legumes | 119 | 6 (5.0%) | 0 | 0 | 6 (5.0%) | 0 | 113 (95.0%) | 113 (95.0%) |
| L. Meats & Substitutes | 863 | 108 (12.5%) | 72 (8.3%) | 0 | 180 (20.9%) | 640 (74.2%) | 43 (5.0%) | 683 (79.1%) |
| M. Miscellaneous | 80 | 10 (12.5%) | 1 (1.3%) | 0 | 11 (13.8%) | 1 (1.3%) | 68 (85.0%) | 69 (86.3%) |
| N. Combination Dishes | 19 | 8 (42.1%) | 9 (47.4%) | 0 | 17 (89.5%) | 0 | 2 (10.5%) | 2 (10.5%) |
| O. Nuts & Seeds | 95 | 3 (3.2%) | 0 | 0 | 3 (3.2%) | 69 (72.6%) | 23 (24.2%) | 92 (96.8%) |
| P. Potatoes | 27 | 3 (11.1%) | 0 | 0 | 3 (11.1%) | 18 (66.7%) | 6 (22.2%) | 24 (88.9%) |
| R. Sauces & Dips | 71 | 29 (40.8%) | 8 (11.3%) | 0 | 37 (52.1%) | 0 | 34 (47.9%) | 34 (47.9%) |
| S. Snacks | 54 | 16 (29.6%) | 8 (14.8%) | 0 | 24 (44.4%) | 2 (3.7%) | 28 (51.9%) | 30 (55.6%) |
| T. Soups | 194 | 141 (72.7%) | 37 (19.1%) | 5 (2.6%) | 183 (94.3%) | 0 | 11 (5.7%) | 11 (5.7%) |
| U. Sugars & Sweets | 102 | 33 (32.4%) | 22 (21.6%) | 0 | 55 (53.9%) | 27 (26.5%) | 20 (19.6%) | 47 (46.1%) |
| V. Vegetables | 385 | 17 (4.4%) | 0 | 0 | 17 (4.4%) | 327 (84.9%) | 41 (10.6%) | 368 (95.6%) |
| W. Foods for <4 years old | 99 | 37 (37.4%) | 8 (8.1%) | 0 | 45 (45.5%) | 0 | 54 (54.5%) | 54 (54.5%) |
| **OVERALL TOTAL** | **3,676** | **844 (23.0%)** | **247 (6.7%)** | **14 (0.4%)** | **1,105 (30.1%)** | **1,579 (43.0%)** | **992 (27.0%)** | **2,572 (69.9%)** |

All values are presented as n (%); n=3,676. ^*^Categories were defined as per Health Canada’s Table of Reference Amounts for Foods (TRA)^(3)^. A total of 354 (9.6%), 62 (1.7%), and 20 (0.5%) products were missing values for total sugars, saturated fats, and sodium, respectively. Abbreviations: TRA, Table of Reference Amounts for Foods.

# Supplementary Table 3. Summary of the total number and proportion of foods that would display a ‘High in’ nutrition symbol by nutrient type based on front-of-pack labelling regulations (*Canada Gazette II)* overall and by Table of Reference Amounts for Foods major category.

|  | **Nutrient-of-concern type  on a ‘High in’ nutrition symbol** | | |
| --- | --- | --- | --- |
| **TRA Category^*^** | **Saturated fat** | **Sugars (total)** | **Sodium** |
| A. Bakery Products | 34 (13.4%) | 37 (14.6%) | 84 (33.1%) |
| B. Beverages | 11 (13.6%) | 36 (44.4%) | 9 (11.1%) |
| C. Cereals & Other Grains | 5 (2.7%) | 9 (4.8%) | 12 (6.4%) |
| D. Dairy Products & Substitutes | 39 (18.8%) | 47 (22.6%) | 24 (11.5%) |
| E. Desserts | 22 (31.4%) | 46 (65.7%) | 11 (15.7%) |
| F. Dessert Toppings & Fillings | 5 (62.5%) | 3 (37.5%) | (0.0%) |
| G. Eggs & Substitutes | 1 (4.8%) | 1 (4.8%) | 1 (4.8%) |
| H. Fats & Oils | 5 (3.5%) | 1 (0.7%) | 33 (22.9%) |
| I. Seafood & Substitutes | 5 (2.0%) | (0.0%) | 19 (7.6%) |
| J. Fruits & Fruit Juices | 1 (0.3%) | 123 (35.9%) | (0.0%) |
| K. Legumes | (0.0%) | (0.0%) | 6 (5.0%) |
| L. Meats & Substitutes | 98 (11.4%) | (0.0%) | 154 (17.8%) |
| M. Miscellaneous | 8 (10.0%) | 1 (1.3%) | 3 (3.8%) |
| N. Combination Dishes | 9 (47.4%) | (0.0%) | 17 (89.5%) |
| O. Nuts & Seeds | 2 (2.1%) | 1 (1.1%) | (0.0%) |
| P. Potatoes | 1 (3.7%) | 1 (3.7%) | 1 (3.7%) |
| R. Sauces & Dips | 10 (14.1%) | 1 (1.4%) | 34 (47.9%) |
| S. Snacks | 17 (31.5%) | 4 (7.4%) | 11 (20.4%) |
| T. Soups | 39 (20.1%) | 11 (5.7%) | 180 (92.8%) |
| U. Sugars & Sweets | 29 (28.4%) | 48 (47.1%) | (0.0%) |
| V. Vegetables | (0.0%) | (0.0%) | 17 (4.4%) |
| W. Foods for <4 years old | 15 (15.2%) | 37 (37.4%) | 1 (1.0%) |
| **OVERALL TOTAL** | **356 (9.7%)** | **407 (11.1%)** | **617 (16.8%)** |

All values are presented as n (%); n=3,676. ^*^*C*ategories were defined as per Health Canada’s Table of Reference Amounts for Foods^(3)^. A total of 354 (9.6%), 62 (1.7%), and 20 (0.5%) products were missing values for total sugars, saturated fats, and sodium, respectively. Abbreviations: TRA, Table of Reference Amounts for Foods.

**REFERENCES**

1. Government of Canada. Regulations Amending Certain Regulations Made Under the Food and Drugs Act (Nutrition Symbols, Other Labelling Provisions, Partially Hydrogenated Oils and Vitamin D). In: Canada Gazette, Part I,152, 6. 2018. Available from: <https://gazette.gc.ca/rp-pr/p1/2018/2018-02-10/html/reg2-eng.html>.

2. Government of Canada. Regulations Amending Certain Regulations Made Under the Food and Drugs Act (Nutrition Symbols, Other Labelling Provisions, Partially Hydrogenated Oils and Vitamin D). In: Canada Gazette, Part II,156, 15. 2022. Available from: <https://canadagazette.gc.ca/rp-pr/p2/2022/2022-07-20/html/sor-dors168-eng.html>.

3. Health Canada. Table of Reference Amounts for Food 2022 [cited 2022 24 November]. Available from: <https://www.canada.ca/en/health-canada/services/technical-documents-labelling-requirements/table-reference-amounts-food/nutrition-labelling.html>.

4. Health Canada. Table of Reference Amounts for Food 2016 [cited 2019 July 15]. Available from: <https://www.canada.ca/en/health-canada/services/technical-documents-labelling-requirements/table-reference-amounts-food.html>.

5. Government of Canada. Food and Drug Regulations. In: CRC, c 870. Ottawa: Government of Canada; 1985. Available from: <https://laws-lois.justice.gc.ca/eng/regulations/c.r.c.,_c._870/page-10.html#h-568846>.
